# Supplementary material for: Thermoresponsive Copolymer Microgels Synthesized via Single‐Step Precipitation Polymerization: Random or Block Structure?
Source: Small. 2025 Oct 9;21(47):e09795. doi: 10.1002/smll.202509795 (PMC12658933; doi:10.1002/smll.202509795)
Supplement: Supplementary file 1 — Supporting Information [file SMLL-21-e09795-s001.pdf]

NANO · MICRO
small

Supporting Information

for *Small*, DOI 10.1002/smll.202509795

Thermoresponsive Copolymer Microgels Synthesized via Single-Step Precipitation Polymerization: Random or Block Structure?

Letizia Tavagnacco, Elena Buratti, Jacopo Vialetto, Francesco Brasili, Elisa Ballin, Kuno Schwärzer, Jitendra Mata, Graziano Di Carmine, Monica Bertoldo, Ester Chiessi, Marco Laurati and Emanuela Zaccarelli**

Supporting Information for: Thermoresponsive Copolymer Microgels Synthesized via Single-Step Precipitation Polymerization: Random or Block Structure?

Letizia Tavagnacco,[†,‡] Elena Buratti,[¶] Jacopo Vialetto,[§,‖] Francesco Brasili,[†,‡] Elisa Ballin,[†,‡] Kuno Schwärzer,[⊥] Jitendra Mata,[#,@] Graziano Di Carmine,[¶] Monica Bertoldo,[△] Ester Chiessi,[▽] Marco Laurati,[*,§,‖] and Emanuela Zaccarelli[*,†,‡]

†*CNR-ISC, Uos Sapienza, Piazzale A. Moro 2, 00185 Roma, Italy*

‡*Dipartimento di Fisica, Sapienza Università di Roma, Piazzale A. Moro 2, 00185 Roma, Italy*

¶*Department of Environmental and Prevention Sciences, University of Ferrara, Via L. Borsari, 46, 44121 Ferrara, Italy*

§*Dipartimento di Chimica "Ugo Schiff", Università di Firenze, Sesto Fiorentino (FI), 50019 Italy*

‖*Consorzio per lo Sviluppo dei Sistemi a Grande Interfase (CSGI), via della Lastruccia 3, Sesto Fiorentino (FI), 50019, Italy*

⊥*Jülich Centre for Neutron Science (JCNS), Forschungszentrum Jülich GmbH, Wilhelm-Johnen-Straße, 52428 Jülich, Germany*

#*Australian Centre for Neutron Scattering (ACNS), Australian Nuclear Science and Technology Organisation (ANSTO), Lucas Height, New South Wales 2234, Australia*

@*School of Chemistry, University of New South Wales, NSW, Australia*

△*Department of Chemical, Pharmaceutical and Agricultural Sciences, University of Ferrara, via L. Borsari 46, 44121 Ferrara, Italy*

▽*Department of Chemical Science and Technologies, University of Rome Tor Vergata, Via della Ricerca Scientifica I, 00133 Rome, Italy.*

E-mail: marco.laurati@unifi.it; emanuela.zaccarelli@cnr.it

Additional Methods

Synthesis of deuterated monomers

NIPAM-d_{10}

All chemicals were used as received. $CHCl_3$, n-hexane and toluene (all absolute or HPLC grade) as well as NaCl were purchased from VWR. D_2SO_4 (98%, 99.5% D) and isopropanol-d_8 (99,5% D) were purchased from Armar Isotopes. $MgSO_4$ (anhydrous) was purchased from Th.Geyer. Acrylonitrile-d_3 (98.7% D) and D_2O (99.90% D) were purchased from Eurisotop.

D_2SO_4 (conc., 50 mL) was cooled to 0 °C in a flask covered with aluminum foil. Then acrylonitrile-d_3 (10.0 g, 11.6 mL, 178 mmol, 1.0 equiv.) was added and the solution was stirred for 5 minutes before slowly adding 2-propanol-d_8 (24.3 g, 27.3 mL, 356 mmol, 2.0 equiv.) via a dropping funnel over 1 hour. The ice bath was removed and the mixture stirred at room temperature for 3 h before pouring it onto 400 mL of ice. The aqueous mixture was slowly neutralized with solid Na_2CO_3, saturated with NaCl and extracted with $CHCl_3$ until TLC analysis (SiO_2, EtOAc) showed, that all of the product had moved into the organic phase. The organic phase was dried over $MgSO_4$, stabilized with a spatula tip of BHT and dried in vacuo. The crude product was then purified via sublimation (55 °C water bath, $2{\cdot}10^{-3}$ mbar vacuum) to yield 16.2 g (131 mmol, 74%) of NIPAM-d_{10}. An additional purification via recrystallization from a mixture of n-hexane and toluene yielded 13.5 g (109 mmol, 61%) of clean product as colorless crystals.

Characterization

The obtained materials were characterized by NMR. NMR spectra were collected on a Varian INOVA 400 MHz spectrometer. All samples were measured at 295 K. Samples were either diluted in $CDCl_3$ (for 1H-NMR) or $CHCl_3$ (for 2H-NMR). 1,2,4,5-Tetrabromobenzene was used as an internal standard to determine the deuteration degree and purity of the products to be > 97%.

NIPMAM-d_{12}

All chemicals were used as received. Isopropanol-d_8 (99.5% D), KBr ($\geq$ 99.0%), potassium phthalimide ($\geq$ 99.0%), DMF (anhydrous, 99.8%), hydrazine monohydrate (98%), MeOH ($\geq$ 99.8%), KOH ($\geq$ 85%), Dichloromethane ($\geq$ 99.9%), 1,2,4,5-tetrabromobenzene (97%) and thionyl chloride ($\geq$ 99%) were purchased from Sigma-Aldrich. H_2SO_4 (95-97%), HCl ($\geq$ 37%) and $MgSO-4$ were purchased from Merck. $CHCl_3$, EtOH, Et_2O, n-hexane and toluene (all absolute or HPLC grade) were purchased from VWR. Methyl methacrylate-d8 (99.5% D) was purchased from Armar Isotopes. The synthesis was composed of the following steps.

Synthesis of Isopropyl Bromide-d_7

Isopropanol-d_8 (100 g, 1.467 mol, M=68.14 g/mol) and KBr (181 g, 1.521 mol, M=119 g/mol) were dissolved in 135 ml H_2O in a 500 ml round bottom flask equipped with a strong magnetic stir bar, a distillation head and a reflux condenser. 198 ml conc. sulfuric acid were added to the reaction mixture at 0 °C via the condenser. After the addition was finished the reaction was heated to 70 °C. Once the temperature in the gas phase had reached 50 °C the product was collected in a 250 ml cooled Schlenk bomb. The bath temperature for the distillation was slowly raised to 90 °C and the reaction was continued until no more product was collected. 190.64 g of a colorless liquid were collected (93.6% crude yield) 2H-NMR showed 5% side product formation. The crude product was used in the next step without further purification.

Synthesis of N-Isopropyl-d_7 Phthalimide

Potassium phthalimide (185.2 g, 1.374 mol 1 eq., M=185.22 g/mol) and crude 2-bromopropane-d_7 (equivalent to 178.5 g, 1.374 mol, M=129.9 g/mol based on 2-bromopropane content of the crude product) were dissolved in 700 ml DMF in a 1 L round bottom flask equipped with a large magnetic stir bar and a reflux condenser. The mixture was stirred for 1 h at 70 °C, 1 h at 120 °C and 2 h at 150 °C. The resulting solution was cooled in a fridge overnight, filtered and the filtrate was washed with 200 ml cold DMF. The organic solutions were combined and the solvent was removed under

reduced pressure to yield the crude product as a yellow solid. Further drying under high vacuum conditions yielded 258.0 g crude product. The crude product was dissolved in 300 ml $CHCl_3$ and filtered for further purification. After removal of the solvent and drying 234.9 g product remained (87.2% chemical yield, 81.7% total yield over 2 steps).

Synthesis of Isopropylamine-d$_7$

N-Isopropyl-d$_7$ phthalimide (20.0 g, 102 mmol, 1.0 equiv) was suspended in 30 mL of methanol. Hydrazine monohydrate (5.2 mL, 107 mmol, 1.05 equiv) was added and the mixture was heated to 80 °C for 1 h. The volatile components were removed in vacuo and the solid residue was cooled to 0 °C and treated with 30 mL of a 7.4 M aqueous KOH solution. The flask was fitted with a Vigreux column and a distillation head and the product was distilled off while slowly heating the oil bath from 75 °C to 110 °C, yielding 5.79 g (87.6 mmol, 86%) of isopropylamine-d$_7$ as a colorless liquid.

Synthesis of Methacrylic Acid-d$_5$

Methyl methacrylate-d$_8$ (13.0 g, 120 mmol, 1.0 equiv) was dissolved in 120 mL of a mixture of DCM and EtOH (9:1). Then 24 mL of a 3 N methanolic KOH solution was added and the reaction was stirred at room temperature for 1 h. The solvent was evaporated in vacuo and the residue was dissolved in water and extracted with DCM and Et2O (1x100 mL each). The aqueous phase was treated with 1 M HCl until the pH reached 2 and then extracted with Et2O (2x) and DCM. The combined organic phases were stabilized by adding hydroquinone (100 mg), dried over MgSO4 and the solvent was evaporated in vacuo (min. 100 mbar, 40 °C) to yield 5.73 g (60 mmol, 50%, containing 0.28 g of EtOH) of methacrylic acid-d$_5$ as a colorless liquid that was used in the next step without further purification.

Synthesis of NIPMAM-d$_{12}$

Crude methacrylic acid (KS058, 60 mmol, 1.0 equiv) was dissolved in 12 mL of DCM alongside 0.2 mL of DMF as a catalyst. Thionyl chloride (4.6 mL, 63 mmol, 1.05 equiv) was added dropwise

and the mixture was stirred under argon for 4 h. In a second flask isopropylamine-d7 (4.1 g, 62 mmol, 1.03 equiv) and triethylamine (17.6 mL, 126 mmol, 2.1 equiv) were dissolved in 120 mL of DCM cooled to 0 °C. Then the crude mixture of acid chloride and DCM was added dropwise. The resulting mixture was stirred over night while slowly warming up to room temperature. Then 100 mL of water were added and the mixture was extracted with DCM (2 x 100 mL). The combined organic phases were washed with 1 M aqueous HCl (2 x 100 mL) and brine (100 mL), dried over $MgSO_4$ and the solvent was evaporated in vacuo. The crude product was recrystallized from n-hexane and toluene to yield 4.5 g (32 mmol, 53%) of NIPMAM-d_{12} as colorless crystals. The deuteration degree was determined by 1H-NMR using 1,2,4,5-tetrabromobenzene as a standard to be >98%.

NMR characterization

NMR spectra were collected on a Bruker Avance III 600 MHz spectrometer, equipped with a Prodigy cryoprobe with a 5 mm PFG AutoX DB probe. All samples were measured at 295 K. Samples were either diluted in $CDCl_3$ (for 1H-NMR) or $CHCl_3$ (for 2H-NMR). We used 1,2,4,5-Tetrabromobenzene as internal standard to determine the deuteration degree and purity of the products to be > 98%.

Additional Results

Swelling curves of P(NIPAM-*co*-NIPMAM) microgels from dynamic light scattering and monomer-resolved simulations

The swelling curves of the P(NIPAM-*co*-NIPMAM) microgels from experiments in H_2O and monomer-resolved simulations are shown in Figure S1 a and b, respectively. The curves are fitted to a sigmoidal function, obtaining $T_{\mathrm{VPT}} = 38.4 \pm 0.1$°C for H-H, $T_{\mathrm{VPT}} = 39.1 \pm 0.1$°C for H-D and $T_{\mathrm{VPT}} = 39.9 \pm 0.1$°C for D-H. The fitting parameters of the simulations are reported in Table S1.

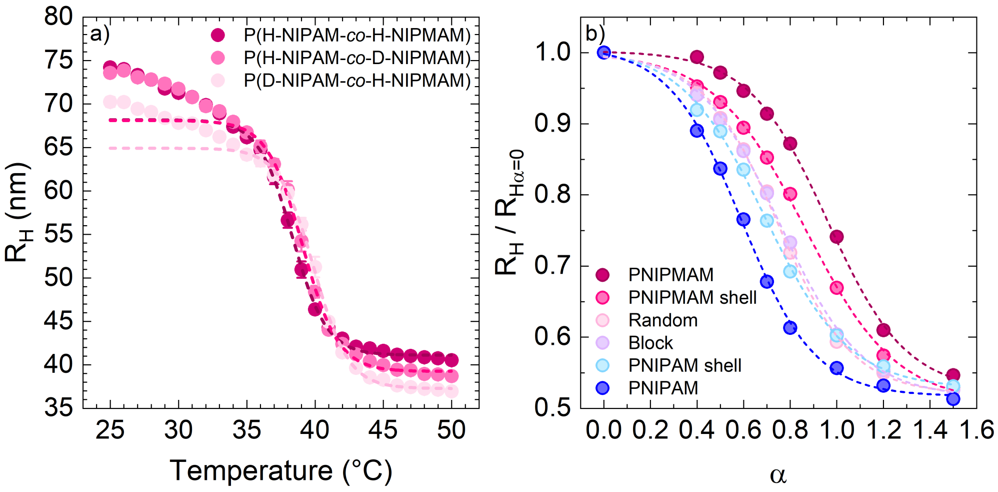

Figure S1: a) Hydrodynamic radius (R_H) from DLS measurements as a function of temperature for H-H (red), H-D (magenta) and D-H (pink). b) Hydrodynamic radius normalized to the corresponding value at the solvophobic parameter $\alpha = 0$ as a function of α for the different microgel topologies: PNIPMAM shell (magenta circles), block (purple circles), random (pink circles), PNIPAM shell (light blue circles), and pure PNIPAM (blue circles) and PNIPMAM (red circles). The dashed lines are sigmoidal fits; for experimental data we set the fit range in proximity of the VPT.

Table S1: T_{VPT} from the sigmoidal fit of the numerical swelling curves.

Microgel topology	α_{VPT}	T_{VPT} / K
PNIPMAM	0.96 (±0.01)	317.0
PNIPMAM shell	0.86 (±0.01)	313.3
Random	0.75 (±0.01)	308.9
Block	0.76 (±0.01)	309.2
PNIPAM shell	0.69 (±0.01)	306.8
PNIPAM	0.59 (±0.01)	303.0

^{1}H-NMR of microgels

To evaluate the copolymer microgels composition, we have employed ^{1}H-NMR experiments. Figures below (Figures S2- S6) show the peaks assignment and the integration in the range between 0.5 and 2.5 ppm, used to calculate the molar composition of the copolymers.

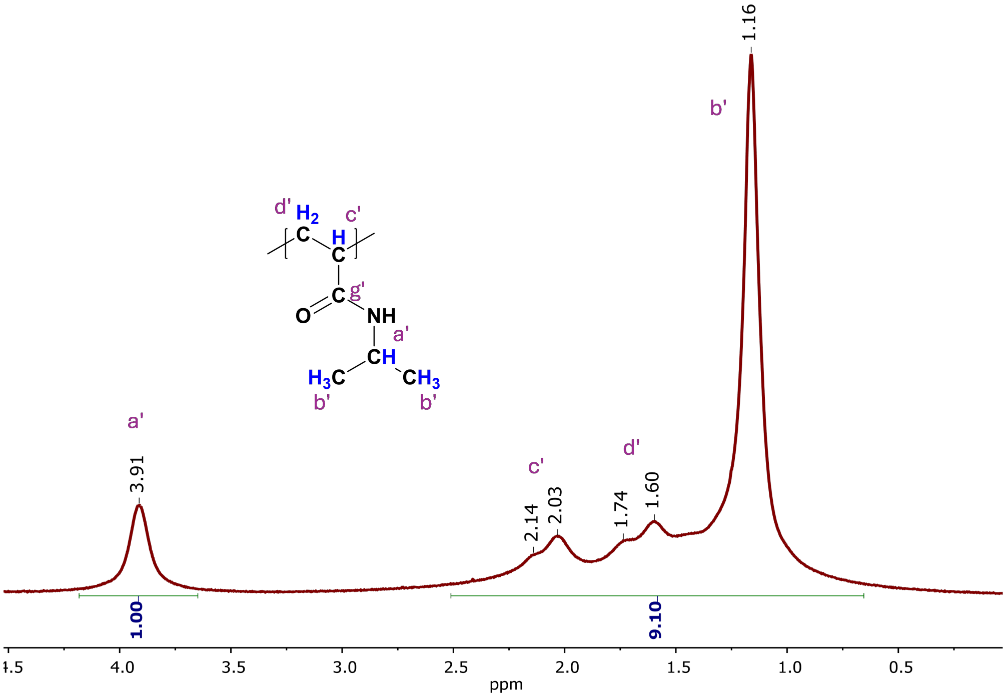

Figure S2: ^{1}H-NMR spectrum at 25°C of PNIPAM microgel dispersion in D_2O.

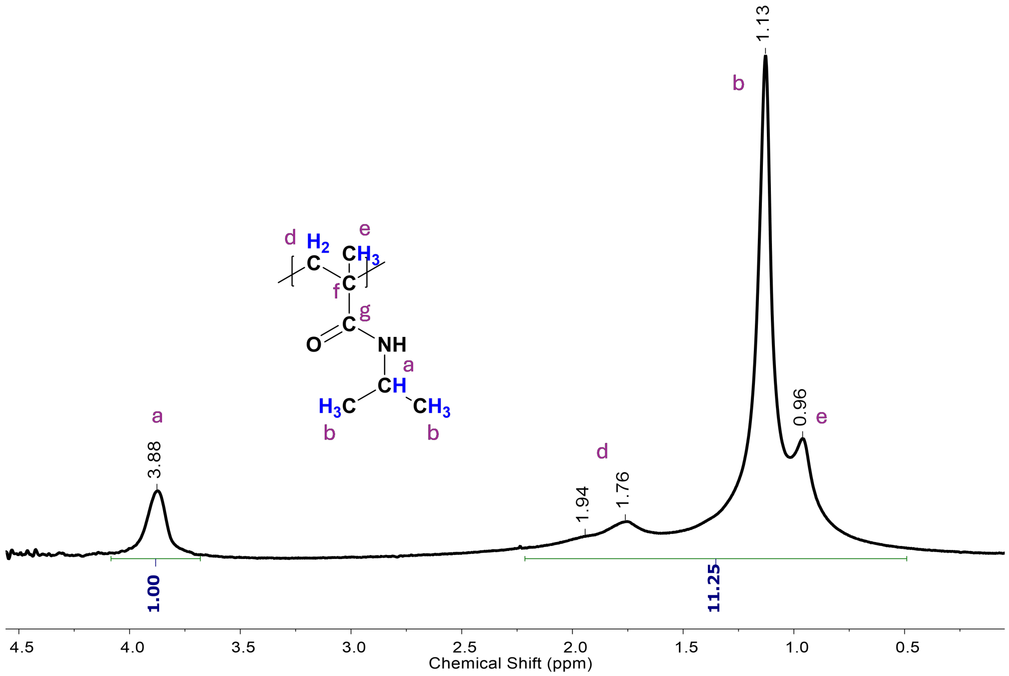

Figure S3: ^{1}H-NMR spectrum at 25°C of PNIPMAM microgel dispersion in D_2O.

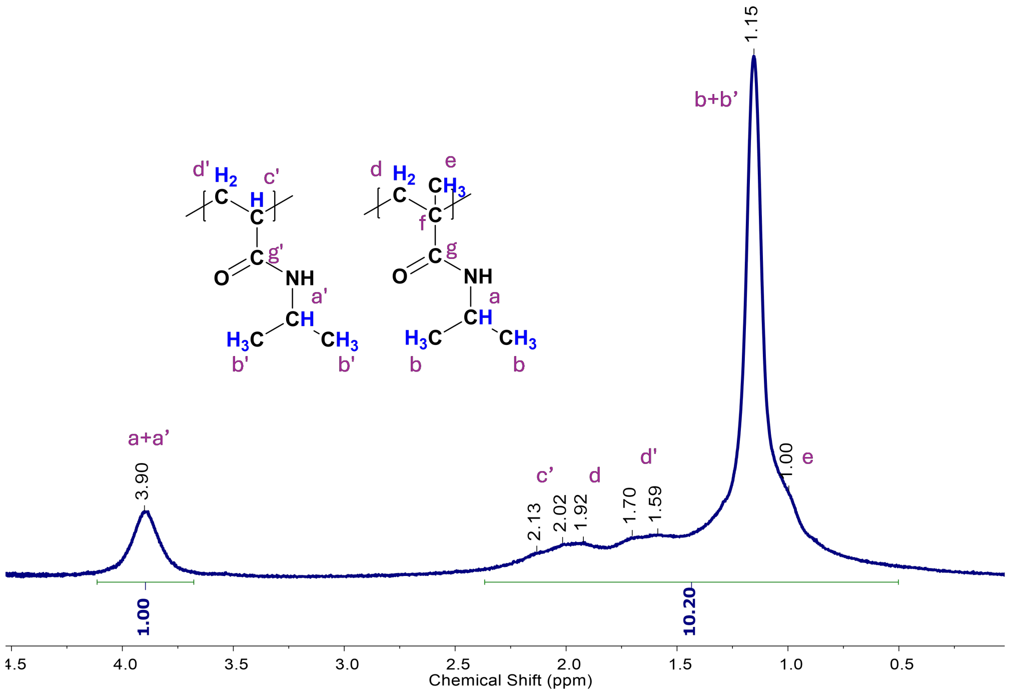

Figure S4: ^{1}H-NMR spectrum at 25°C of P(H-NIPAM-*co*-H-NIPMAM) microgel dispersion in D_2O.

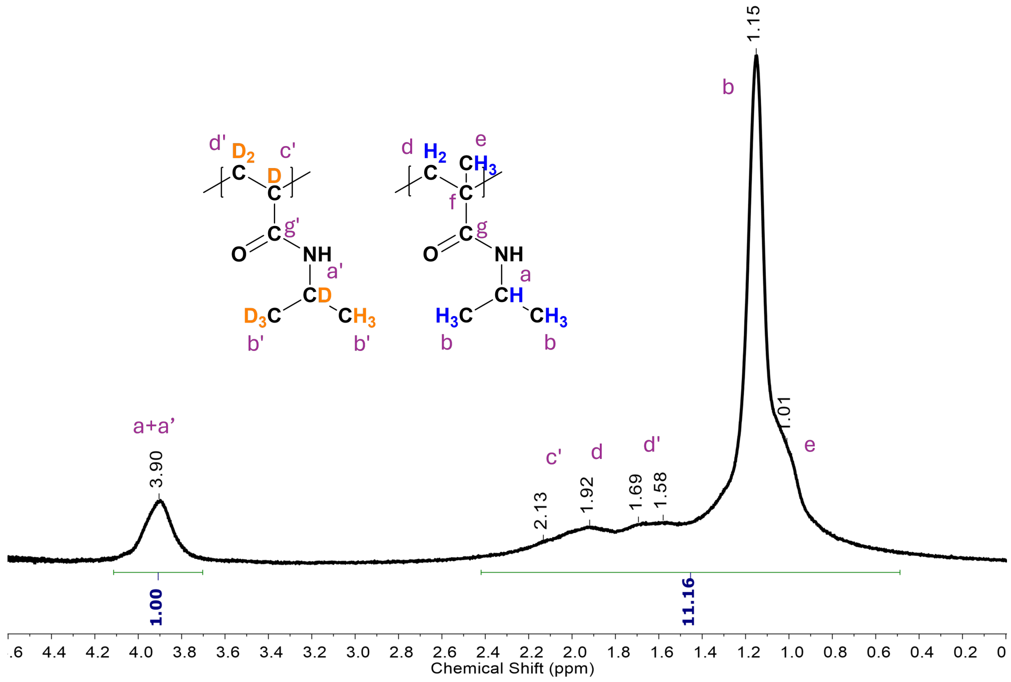

Figure S5: ^{1}H-NMR spectrum at 25°C of P(D-NIPAM-*co*-H-NIPMAM) microgel dispersion in D_2O. The D-NIPAM is a mixture of 32% mol of NIPAM and 68% mol of NIPAM-d_{10}

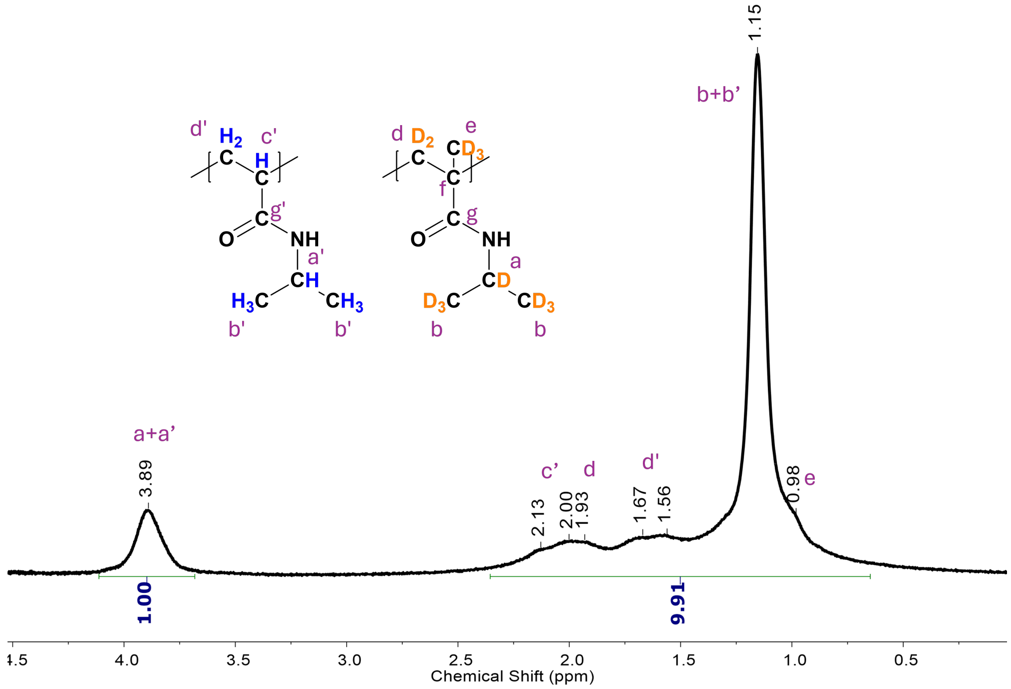

Figure S6: ^{1}H-NMR spectrum at 25°C of P(H-NIPAM-*co*-D-NIPMAM) microgel dispersion in D_2O. The D-NIPMAM is a mixture of 44% mol of NIPMAM and 56% mol di NIPMAM-d_{12}

^{13}C-NMR of microgels

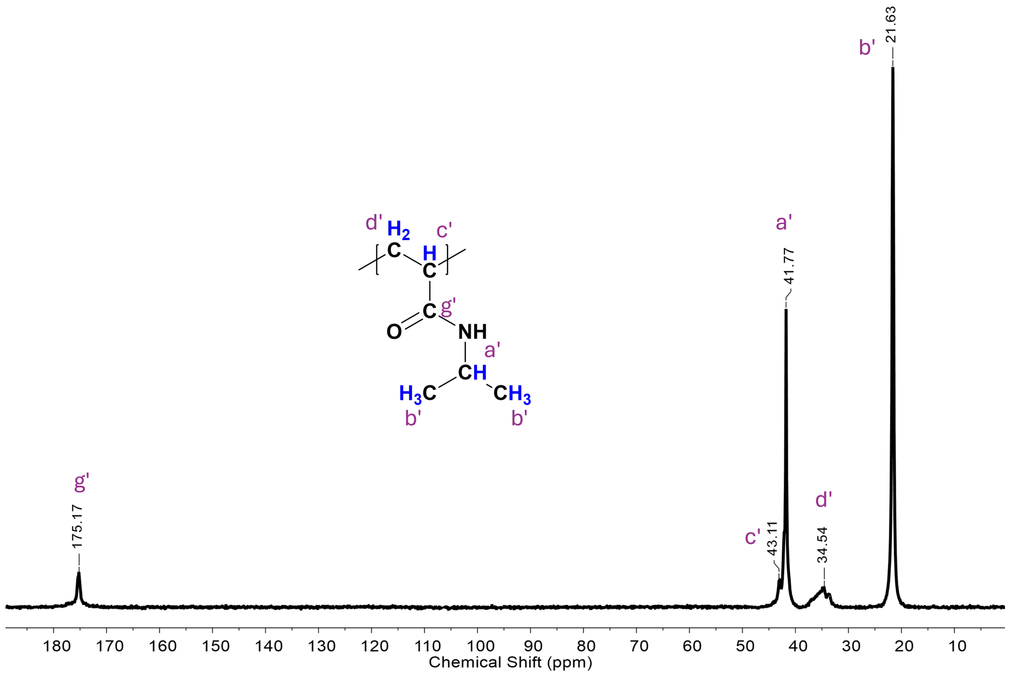

Figure S7: ^{13}C-NMR spectrum at 25°C of PNIPAM microgel dispersion in D_2O.

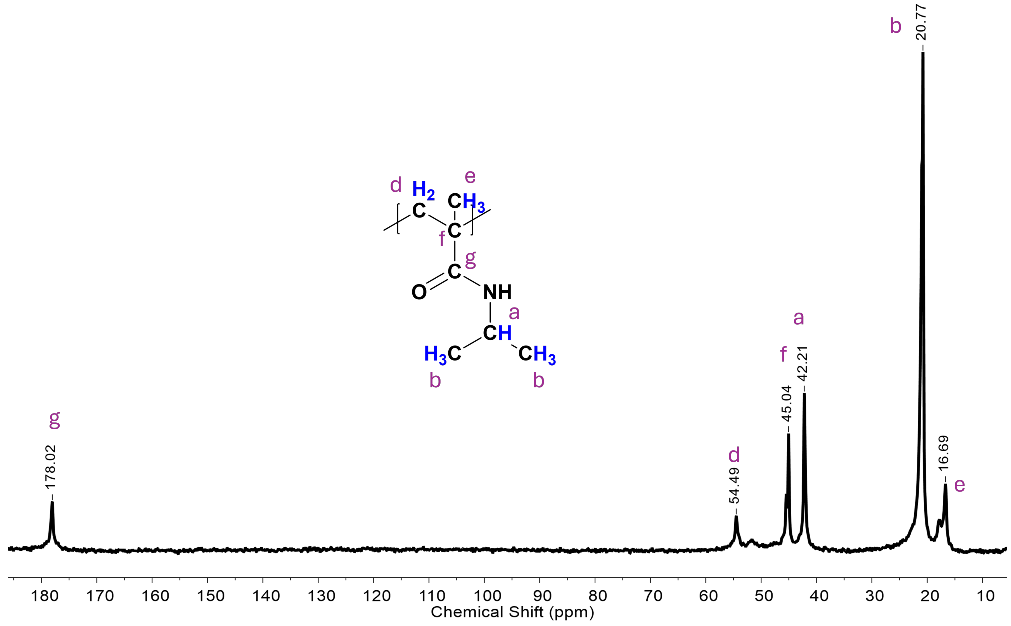

Figure S8: ^{13}C-NMR spectrum at 25°C of PNIPMAM microgel dispersion in D_2O.

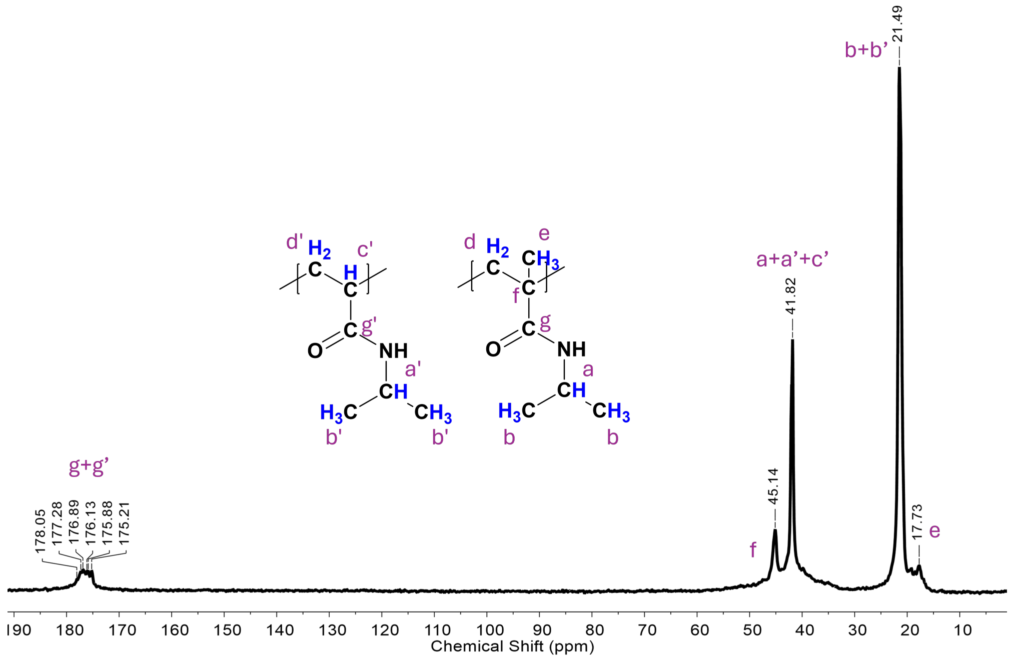

Figure S9: ^{13}C-NMR spectrum at 25°C of P(H-NIPAM-*co*-H-NIPMAM) microgel dispersion in D_2O.

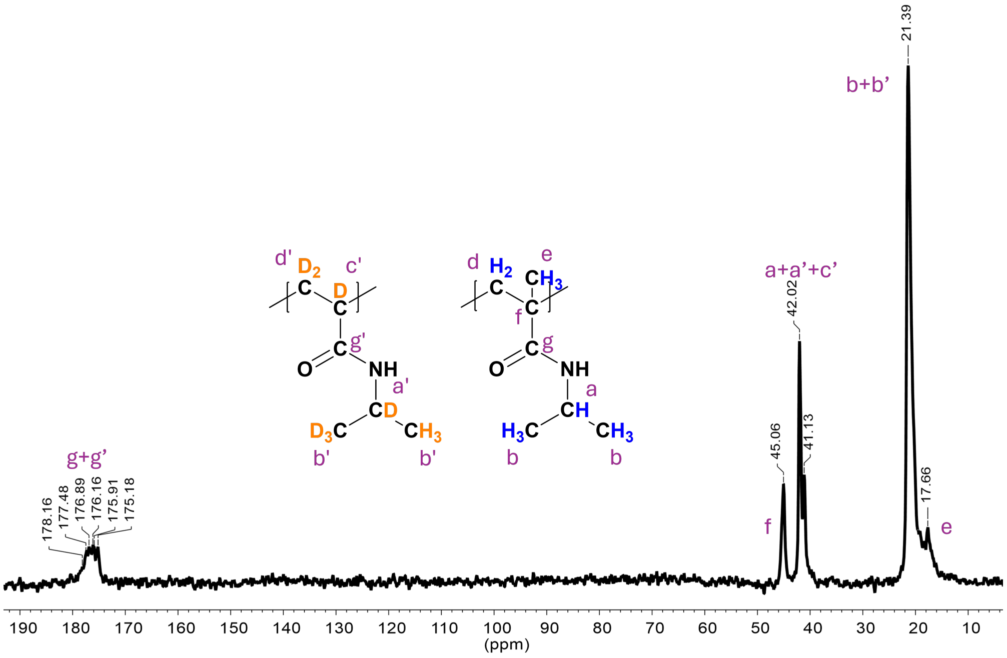

Figure S10: ^{13}C-NMR spectrum at 25°C of P(D-NIPAM-*co*-H-NIPMAM) microgel dispersion in D_2O. The D-NIPAM is a mixture of 32% mol of NIPAM and 68% mol of NIPAM-d_{10}

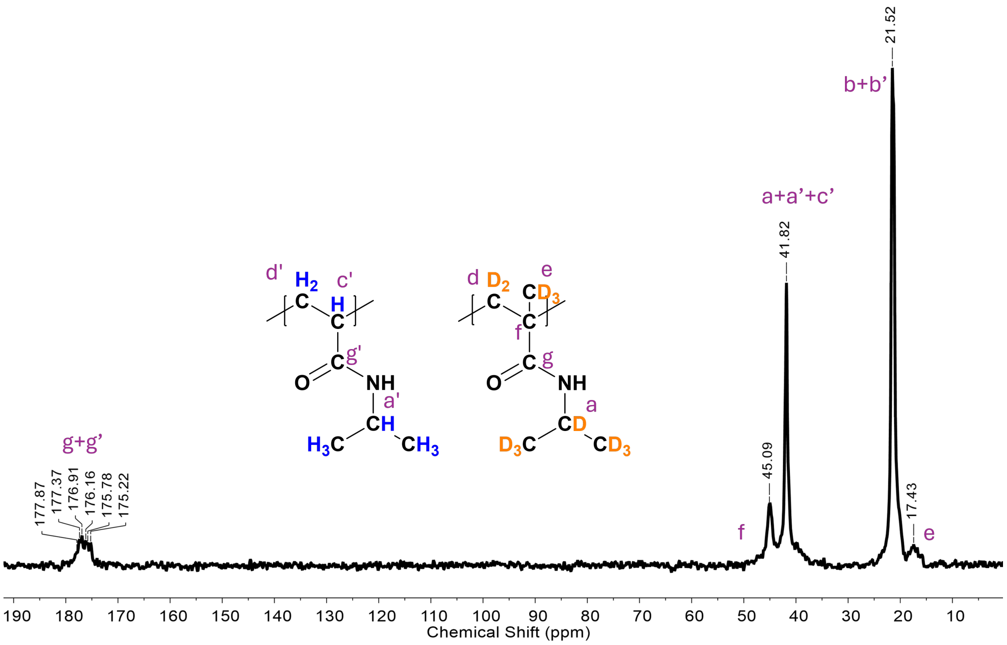

Figure S11: ^{13}C-NMR spectrum at 25°C of P(H-NIPAM-*co*-D-NIPMAM) microgel dispersion in D_2O. The D-NIPMAM is a mixture of 44% mol of NIPMAM and 56% mol di NIPMAM-d_{12}

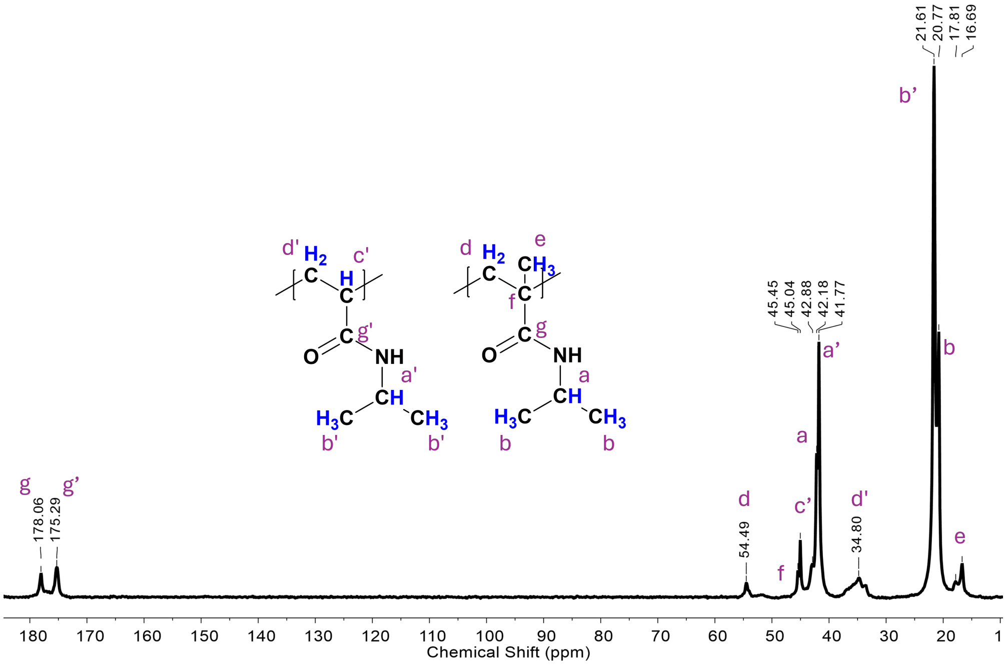

Figure S12: ^{13}C-NMR spectrum at 25°C of mixture (1/1 by weight) of PNIPAM and PNIPMAM microgels

Carbonyl signal deconvolution in the ^{13}C-NMR spectra

Deconvolution of the carbonyl signal was used to determine the relative percentage contribution of the different sequences (PPP, PPM, MPM, MMM, MMP, and PMP) to the overall peak. In the images shown below (Figure S13- S16), the black line represents the original acquired spectrum, the blue lines are the deconvoluted single peaks, the violet line is the sum of the deconvoluted peaks, almost superimposed with the original spectrum, and the red line is the difference between the original spectrum and the deconvoluted one.

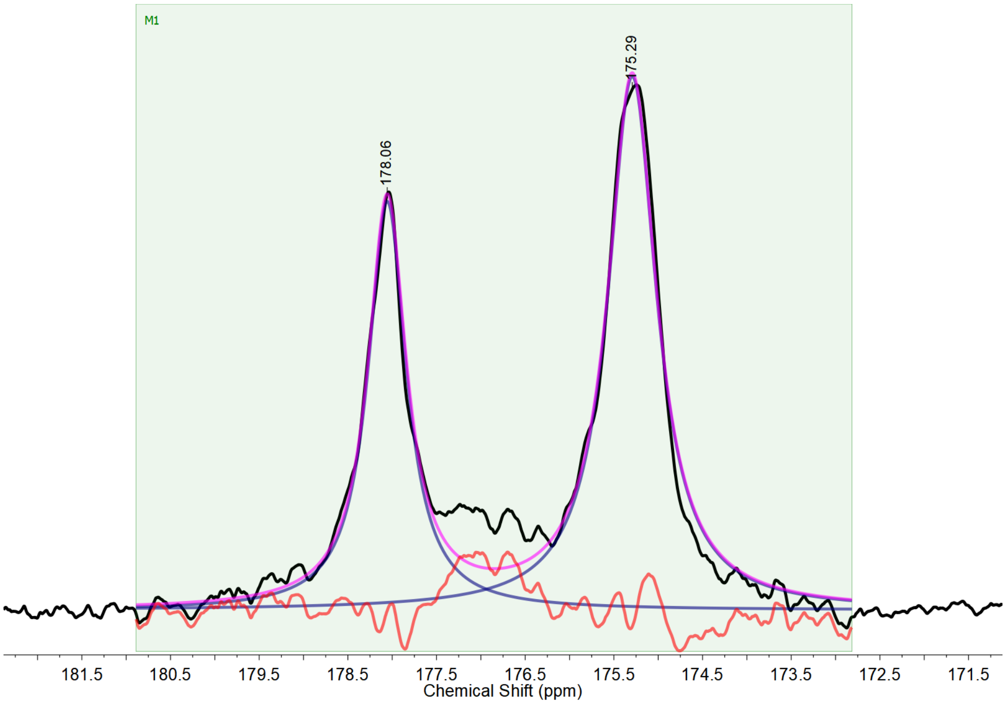

Figure S13: Deconvolution of the carboxylic peaks in the ^{13}C NMR spectrum of mixture of PNIPAM and PNIPMAM microgels

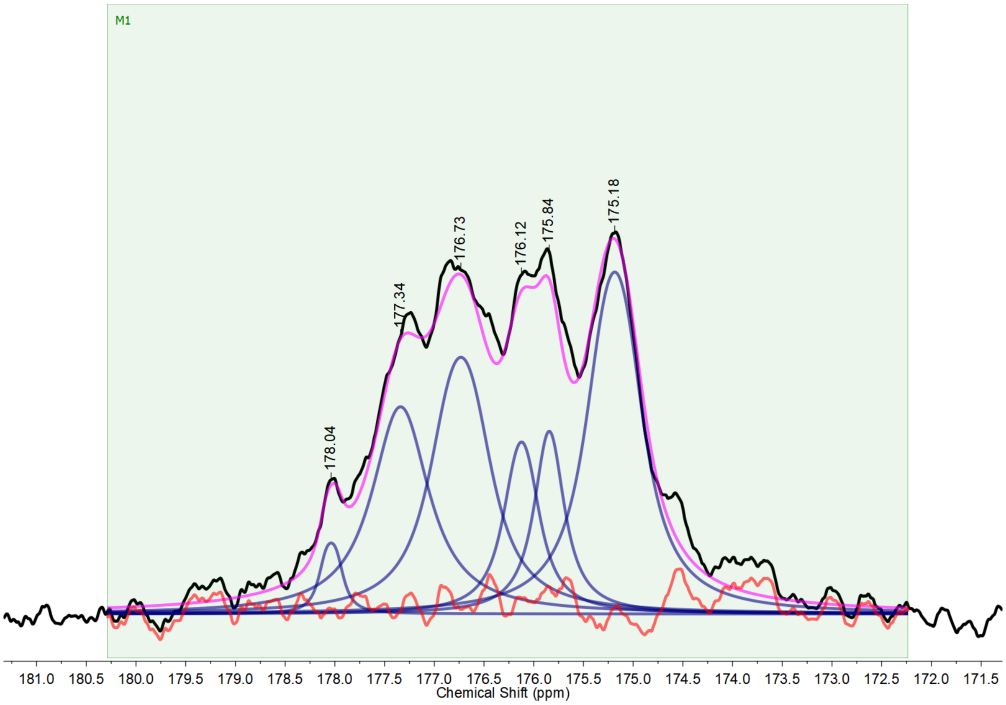

Figure S14: Deconvolution of the carboxylic peaks in the ^{13}C NMR spectrum of P(H-NIPAM-*co*-H-NIPMAM) microgel

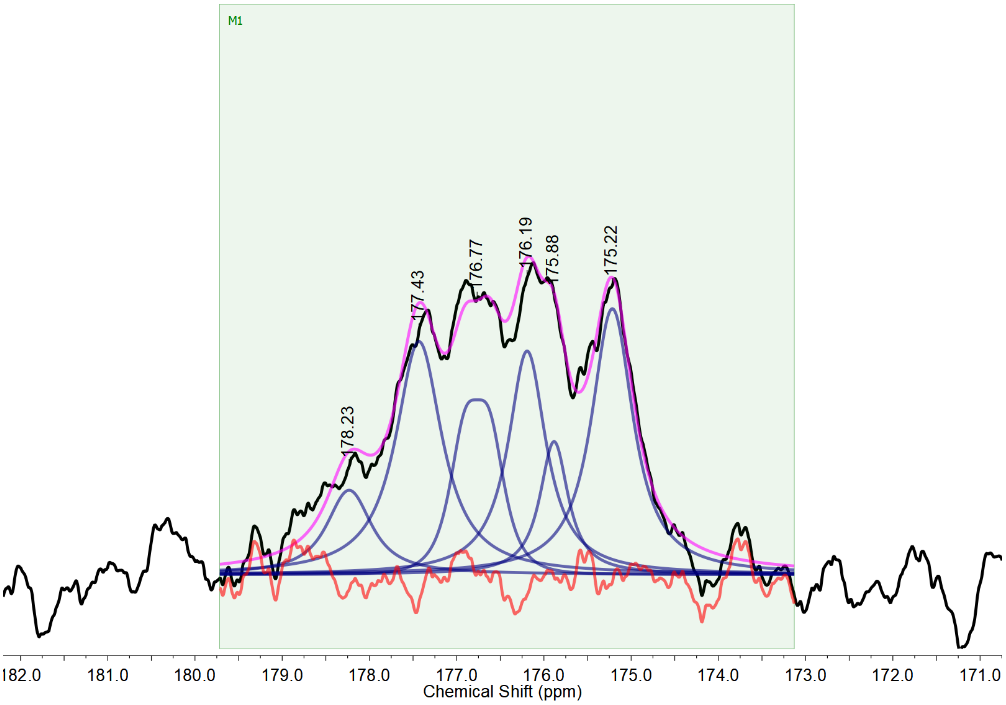

Figure S15: Deconvolution of the carboxylic peaks in the ^{13}C NMR spectrum of P(D-NIPAM-*co*-H-NIPMAM) microgel

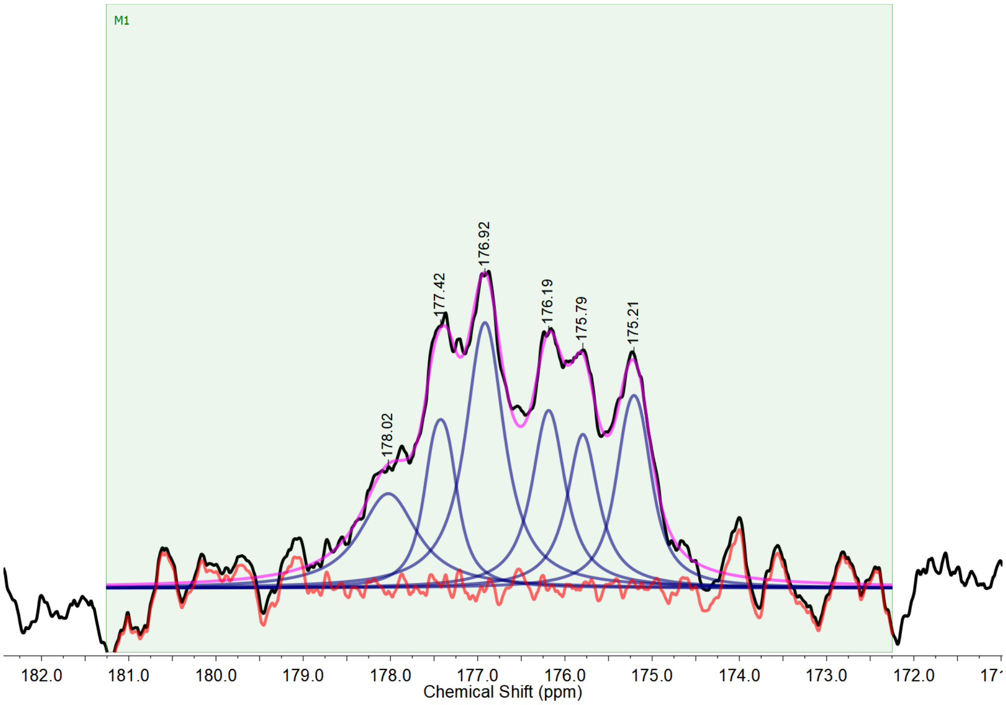

Figure S16: Deconvolution of the carboxylic peaks in the ^{13}C NMR spectrum of P(H-NIPAM-*co*-D-NIPMAM) microgel

Small-angle neutron scattering fit parameters

Small-angle neutron scattering data of all microgels systems were fitted by using a fuzzy sphere model.[S1] The fitting results well reproduce the experimental data, over the entire explored q-range. These results reveal that all copolymer microgels have fuzzy surfaces and dense cores. The fitted parameters are listed in Tab. S2.

Table S2: Parameters obtained by fits of the experimental SANS intensities measured for dilute microgel suspensions using Eq. 1 together with Eq. 2 for the form factor $P(q)$. The last column reports the polydispersity obtained by considering a Schulz distribution of the radius. H-H, H-D and D-H indicated P(H-NIPAM-*co*-H-NIPMAM), P(H-NIPAM-*co*-D-NIPMAM) and P(D-NIPAM-*co*-H-NIPMAM), respectively.

	T (°C)	R (nm)	σ (nm)	ζ (nm)	poly
H-H	20	43.5 ± 2	9.5 ± 0.5	6 ± 0.4	0.15
	35	38.0 ± 1.0	8.0 ± 0.5	8.0 ± 0.5	0.15
	37.5	34.0 ± 0.8	6.5 ± 0.4	8.5 ± 1.0	0.14
	50	29.0 ± 0.6	0.5 ± 0.1	0.5 ± 0.2	0.14
H-D	20	44.0 ± 1.0	10.0 ± 1.0	6.5 ± 0.5	0.16
	35	40.0 ± 1.0	8.5 ± 0.7	7.0 ± 0.6	0.15
	37.5	39.0 ± 1.2	8.3 ± 0.4	8.0 ± 0.5	0.15
	50	29.0 ± 0.6	2.0 ± 0.2	4.0 ± 0.3	0.14
D-H	20	46.0 ± 2.0	10.0 ± 1.0	7.5 ± 0.8	0.15
	35	40.0 ± 1.5	8.5 ± 0.6	8.5 ± 0.5	0.15
	37.5	37.0 ± 1.5	7.5 ± 0.5	8.8 ± 0.8	0.14
	50	28 ± 1.0	3.5 ± 0.3	4.5 ± 0.5	0.14

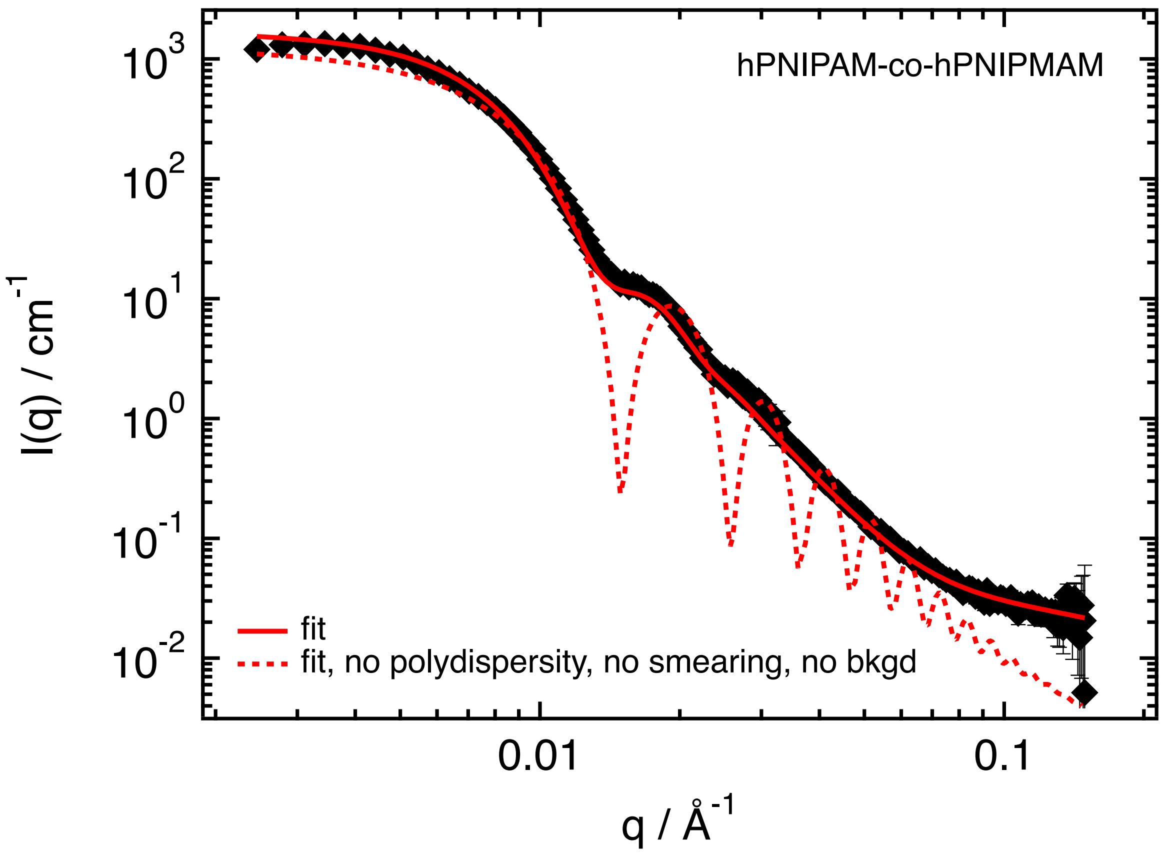

Figure S17: Form factor at 20°C of P(H-NIPAM-*co*-H-NIPMAM) microgels including fit with equation Eq. 2 including resolution smearing and polydispersity (solid line) and after removal of resolution smearing and polydispersity (dashed line).

Small block topology of P(NIPAM-*co*-NIPMAM) microgels

The *block* topology model used in this study was designed to provide a description of an extreme case of microgel internal architecture, exhibiting local domain structures. We defined domains consisting of polymer segments located between two cross-linker particles in order to generate sufficiently large regions. To further investigate the role of domain size, we developed an additional topology, referred to as *small block* (Figure S18). This model was generated by randomly distributing blocks containing roughly ten consecutive PNIPMAM monomers onto a PNIPAM microgel network. The comparison between experimental and simulated form factors is shown in Figure S19. These findings reveal that the *small block* topology also provides a satisfactory description of the experimental SANS data, thus supporting the hypothesis that preferential formation of local domain structures occurs within the microgel network.

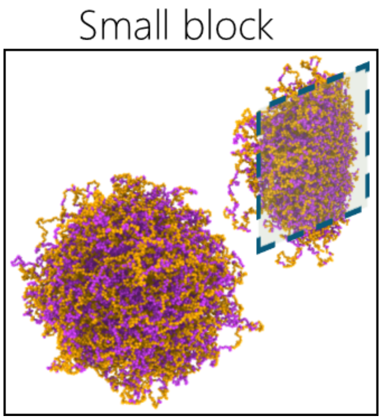

Figure S18: Representative snapshot from the monomer-resolved simulations showing a small block microgel topology. PNIPAM and PNIPMAM particles are shown in purple and yellow, respectively. Cross-linkers are shown in purple. The inner part of the microgels structures is also highlighted with a sliced representation on the top panels.

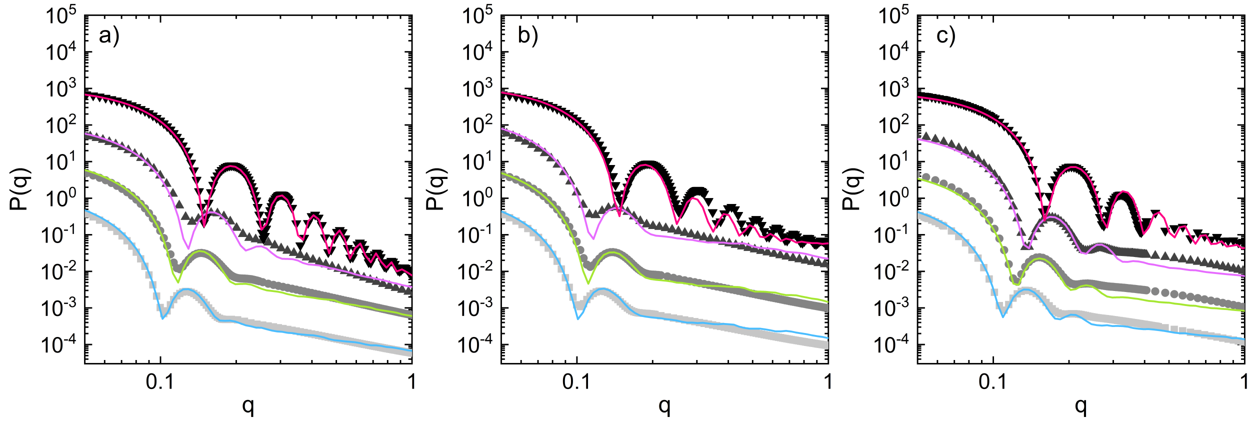

Figure S19: Comparison between the experimental form factors (P(q)) measured for (a) P(H-NIPAM-*co*-H-NIPMAM), (b) P(H-NIPAM-*co*-D-NIPMAM), and (c) P(D-NIPAM-*co*-H-NIPMAM) microgels and the numerical one calculated for the *small block* topology. Experimental data are measured at 20°C (light gray squares), 35°C (gray circles), 37.5°C (dark gray triangles), and 50°C (black diamonds) and compared to numerical data calculated at the corresponding α values used for the other model topologies. Data at different T values are vertically shifted for clarity.

Polymer-water hydrogen bonding analysis

The occurrence of polymer-water hydrogen bond was determined by using the geometric criteria of an acceptor-donor distance lower than 0.35 nm and a hydrogen-donor-acceptor angle lower than 30°. To account for the effect of the local environment, polymer-water hydrogen bonds were calculated for each polymer repeating unit and averaged over all repeating units of the different chain models showing the same environment.

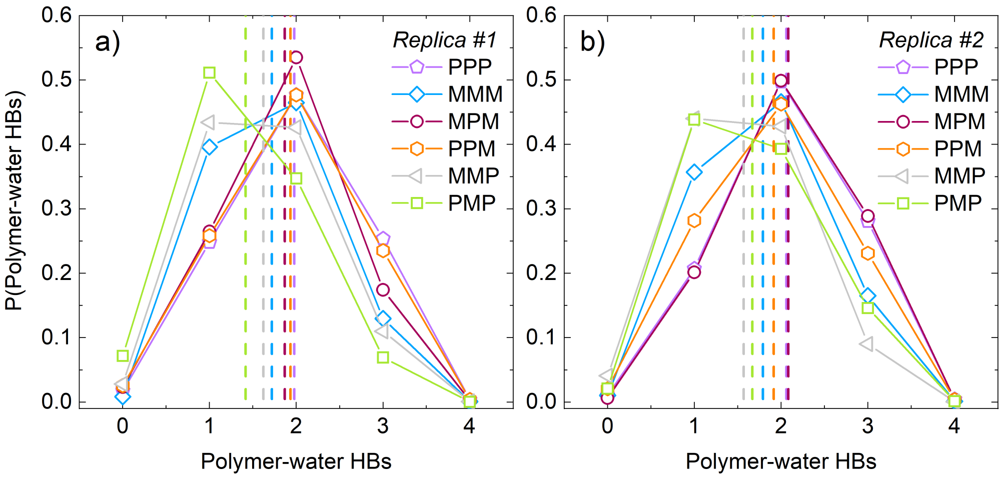

Figure S20: Probability distribution of the number of polymer-water hydrogen bonds formed by each repeating unit in the atomistic chains as a function of the neighbouring repeating units: PPP (purple pentagons); MMM (blue diamonds); MPM (red circles); MPP (orange hexagons); MMP (gray triangles); and PMP (green squares). Data are calculated at 298 K and averaged over all model systems for a) the first and b) the second replica. Vertical lines are averaged distribution values.

Specifically, the probability distribution of polymer–water hydrogen bonds for each NIPAM (denoted as P) and NIPMAM (denoted as M) repeating unit was classified on the basis of their neighboring repeating units in six types: (PPP) a PNIPAM unit in between two PNIPAM units; (MMM) a PNIPMAM unit in between two PNIPMAM; (MPM) a PNIPAM unit in between two PNIPMAM units; (MPP) a PNIPAM unit in between a PNIPAM and a PNIPMAM unit; (MMP) a PNIPMAM unit in between a PNIPAM and a PNIPMAM unit; and (PMP) a PNIPMAM unit in between two PNIPAM units. The PNIPAM and PNIPAMAM chains contain 88 PPP and 88 MMM configurations, respectively. The block chain consists of 43 PPP, 43 MMM, 1 MMP, and

1 MPP configurations. The random chain is composed by 7 PPP, 22 PPM, 14 PMP, 6 MMM, 24 MMP, and 15 MPM configurations. The analysis was carried out over the last 500 ns of trajectory data, sampled every 100 ps. The probability distribution of the number of polymer-water hydrogen bonds was calculated for two independent replicas, as shown in Figures S20a and S20b, which confirm the robustness of results.

Mapping temperature in coarse-grained simulations

The different volume phase transition temperature of PNIPAM and PNIPMAM microgels is accounted into the *in silico* model by assigning a different value of α to each monomer, based on the relation between α and temperature determined for PNIPAM microgels in Ref. S2. For mixed interactions occurring between PNIPAM and PNIPMAM monomers the average value of α is used. Figure S21a summarizes the temperature mapping used to model the monomer interactions in this study. To compare experimental and numerical form factors and reproduce the temperature-dependent evolution of the experimental form factors, we adjust the value of the solvophobic parameter α, keeping consistent across the different microgel topologies. The resulting mapping between α and temperature is reported in Figure Figure S21b.

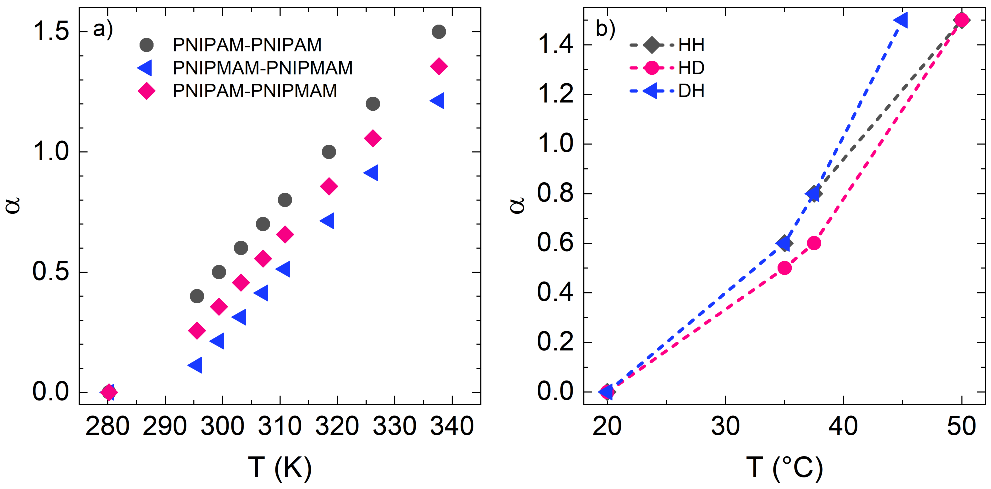

Figure S21: a) Values of the solvophobicity parameter α employed to describe PNIPAM-PNIPAM, PNIPMAM-PNIPMAM and mixed interactions at each temperature. b) α-temperature relation for H-PNIPAM-co-H-PNIPMAM (black diamonds), H-PNIPAM-co-D-PNIPMAM (magenta circles), and D-PNIPAM-co-H-PNIPMAM (blue diamonds) microgels.

References

[S1] Stieger, M.; Richtering, W.; Pedersen, J. S.; Lindner, P. Small-angle neutron scattering study of structural changes in temperature sensitive microgel colloids. *The Journal of chemical physics* **2004**, *120*, 6197–6206.

[S2] Gnan, N.; Rovigatti, L.; Bergman, M.; Zaccarelli, E. In silico synthesis of microgel particles. *Macromolecules* **2017**, *50*, 8777–8786.
